# Tacrolimus-induced cholestatic hepatotoxicity after renal transplantation: a case report

**DOI:** 10.1186/s13256-024-04394-6

**Published:** 2024-02-26

**Authors:** Fatemeh Pourrezagholi, Hossein Amini, Omid Moradi, Shadi Ziaie

**Affiliations:** 1 Chronic Kidney Disease Research Center, Shahid Beheshti University of Medical Sciences, Tehran, Iran; 2 Department of Clinical Pharmacy, School of Pharmacy, Shahid Beheshti University of Medical Sciences, Tehran, 35113-19968 Iran; 3 Department of Clinical Pharmacy, Faculty of Pharmacy, Hormozgan Unviversity of Medical Sciences, Hormozgan, Iran

**Keywords:** Tacrolimus, Hepatotoxicity, Hyperbilirubinemia

## Abstract

**Background:**

In this manuscript, we report a case of tacrolimus-associated hepatotoxicity in a kidney transplant recipient.

**Case presentation:**

In this case report, a 56 years old Arab male patient who received a kidney transplant presented with icterus, weakness, and lethargy two weeks after transplantation and tacrolimus initiation. In laboratory analysis hyperbilirubinemia and a rise in hepatic enzymes were observed. All possible causes of hepatotoxicity were examined. The panel for infectious causes was negative. Drug-induced liver injury was diagnosed. The patient’s immunosuppressive regimen was changed to a cyclosporine-based regimen and after this change bilirubin and hepatic enzymes decreased and the patient was discharged without signs and symptoms of hepatitis.

**Conclusion:**

It seems that the patient’s hyperbilirubinemia was due to tacrolimus, and the patient’s bilirubin decreased after stopping tacrolimus.

## Background

Calcineurin inhibitors, including tacrolimus and cyclosporine, are widely used in kidney transplantation. So far, there is limited information on the hepatotoxicity of tacrolimus, and few studies have reported liver damage from tacrolimus, which could be through the cholestatic pattern [1]. We report a case of cholestatic pattern hepatotoxicity with tacrolimus, which was partially resolved by stopping tacrolimus.

## Case presentation

A 56-year-old Arab male patient from Iraq, with diagnosis of end-stage renal disease due to hypertension has been referred to receive a kidney transplant. Before the transplant, the patient underwent hemodialysis for two years through the brachial fistula of the left hand 3 times a week for 3 hour each time. The patient had no history of any special disease except for uncontrolled hypertension, and the patient’s habitual history was not remarkable. Before the kidney transplantation surgery, ultrasonography of the abdomen and pelvis were done, and the patient’s liver was found normal in size and shape. The intrahepatic and extrahepatic bile ducts were not dilated. The port system had normal size and appearance, and no stone or space-occupying lesion was observed in the gall bladder. Also, echocardiography, endoscopy, and pulmonary function tests were performed based on the institute protocol, which did not have any specific clinical abnormality. The patient underwent the surgery (day 0) and was discharged twelve days later (day 12) in a stable condition with medication instructions as followed. Tacrolimus (Prograf, *Janssen*) 1.5 mg twice daily, prednisolone 10 mg daily, mycophenolate mofetil 500 mg twice daily, cotrimoxazole 480 mg daily, valganciclovir 450 mg twice daily, calcitriol 0/25 micg daily, epoetin alfa 10,000 IU three times weekly, folic acid 1 mg daily, calcium carbonate 500 mg daily, famotidine 20 mg twice daily, valsartan 80 mg twice daily, carvedilol 6/25 mg twice daily and prazosin 2/5 mg twice daily. The patient’s lab data at the time of discharge are listed in Table 1.

**Table 1:** Laboratory data of the patient in the first and second discharge

Laboratory data	Day 12 (First discharge)	Day 40 (second discharge)
WBC (10^3^ cells/μL)	12.1	10.4
Hb (g/dL)	8.5	10.3
Plt (10^3^/μL)	218	374
Serum creatinine (mg/dL)	1.1	0.7
AST (U/I)	22	36
ALT (U/I)	26	25
ALP (U/I)	278	270
Bilirubin, total (mg/dL)	1.64	5.45
Bilirubin, direct (mg/dL)	0.78	2.38
PT (seconds)	15	14.3
PTT (seconds)	27	28
INR	1.25	1.19

WBC: white blood cell, Hb: hemoglobin, Plt: platelet, AST: aspartate aminotransferase, ALT: alanine transaminase, ALP: alkaline phosphatase, INR: international normalized ratio, PT: Prothrombin time, PTT: partial thromboplastin time

**Fig. 1:**
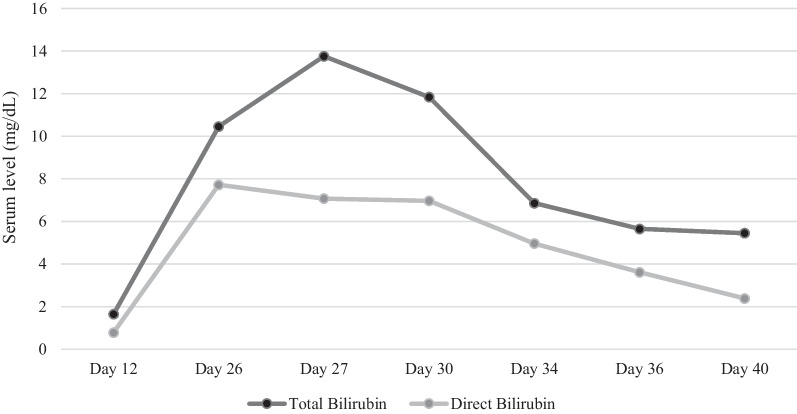
Trend of total and direct bilirubin of the patient

Two weeks after discharge (day 26), the patient presented with complaints of weakness, lethargy, decreased urine volume, icterus. In laboratory results pancytopenia was found (WBC 0.9 × 10^3^ cells/μL, Hb7.9 g/dL, Plt 112 × 10^3^ cells/μL). In the examination of the head and neck, the sclera was icteric, the pupils were reactive to light, the mucous membranes were dry, the jugular vein was not prominent and the thyroid was of normal size and consistency. No petechiae, rashes, or ecchymoses were observed. No organomegaly was observed in the physical examination of the abdomen. The neurologic, cardiac and pulmonary examinations were normal. The patient’s liver enzymes on admission include serum aspartate aminotransferase (AST) 45 IU/I, alanine transaminase (ALT) 40 IU/I, alkaline phosphatase (ALP) 400 IU/I. Bilirubin total and direct were 10.46 and 7.72 mg/dL respectively. Viral profiles including hepatitis B virus (HBV), hepatitis C virus (HCV), hepatitis A virus (HAV), cytomegalovirus (CMV), coronavirus disease- 2019 (COVID-19), influenza, and blood and urine cultures were negative. Abdominal and pelvic ultrasonography were requested for the patient, and the liver was reported normal in size and shape, without space-occupying lesions. The intrahepatic and extrahepatic bile ducts were not dilated and the portal vein was seen with an increased diameter. Gallbladder has normal dimensions and shape, with normal thickness in wall, and no stones or space-occupying lesions were observed. The patient’s coagulation tests were slightly elevated (INR 1.62, PTT 28 seconds). Therapeutic drug monitoring for tacrolimus reported 6.2 ng/dL serum level. According to the results of work-ups based on the timeline of the patient’s symptoms appearance, the medical team suspected drug-induced hyperbilirubinemia, and after examining the patient’s medications, the most suspected was tacrolimus. Therefore, mycophenolate and tacrolimus were stopped (day 27) for the patient, and intravenous hydrocortisone and ursodeoxycholic acid were prescribed. The patient’s serum creatinine was normalized with fluid therapy and corticosteroids, and cyclosporine 100 mg twice daily (day 30) plus mycophenolate 500 mg twice daily (day 35) was started for the patient. The patient’s liver tests decreased and the patient was discharged with AST 36 U/I, ALT 25 U/I, ALP 270 U/I, bilirubin total 5.45 mg/dL and bilirubin direct 2.38 mg/dL. The trend of the total and direct bilirubin is shown in Fig. 1. In the follow up period due to an episode of infection the patient was hospitalized in another medical center and unfortunately expired.

## Discussion and conclusion

Calcineurin inhibitors (CNIs), including tacrolimus and cyclosporine, are widely used as major immunosuppressants in kidney transplantation. Hepatotoxicity was reported with both agents as a rare adverse reaction [2]. The exact mechanism of tacrolimus hepatotoxicity is not completely understood, but it can be due to idiosyncratic metabolic or immunologic reactions [3]. In vivo studies revealed that tacrolimus can reduce the biliary excretion of glutathione and, to a lesser extent, bicarbonate, thereby causing hepatic cholestasis [4]. In a study of three kidney transplant recipients, receiving tacrolimus, biopsy proven venous occlusive disease (VOD), was developed a few weeks after transplantation [5]. In our patient, injury caused by ischemia, infection, vascular and biliary complications was rolled out. Also, due to not stopping corticosteroids and restarting mycophenolate after a few days, and decreasing the patient’s bilirubin after tacrolimus de-challenge and starting cyclosporine, the only clinical cause of hyperbilirubinemia was tacrolimus.

In a case–control study, investigating 1010 kidney transplant patients, 90 patients suffered liver damage. Tacrolimus was administered in all 90 patients. the pattern of the hepatotoxicity was reported cholestatic in most of them [68 patients (75.6%)] followed by 16 patients with hepatocellular pattern and 6 patients showed mixed damage. Mycophenolate was received in 88 patients as part of the immunosuppressant regimen. The average day of occurrence of cholestatic complications was 15 days, similar to our case [6]. In a case report, a 28-year-old patient presented 5 months after receiving a kidney transplant, with a bilirubin of 5.1 mg/dL, which reached 12.5 mg/dL after one week of hospitalization. All clinical workups were normal. The tacrolimus level was requested for the patient, which was equal to 6.1 ng/dL, but within a week, the patient’s bilirubin rose again. By tacrolimus discontinuation, bilirubin started to decrease in the first week and returned to normal after 8 weeks [7].

As in this study, a liver biopsy was part of our treatment plan, but due to the decreasing trend of the patient’s bilirubin, a liver biopsy was not performed. Also, the level of tacrolimus in this study was close to our study, which can suggest that this complication can probably occur at the therapeutic level, as in a study conducted by Rainer Ganschow *et al.* among 112 children who underwent liver transplantation, 6 patients had raised bilirubin and in all 6 patients, tacrolimus level was below 15 ng/dL, and by converting tacrolimus to cyclosporine, the cholestasis of all patients was resolved [8].

We concluded that the patient’s hepatotoxicity was caused by tacrolimus because no other cause was identified by diagnostic evaluations and the patient’s bilirubin decreased after stopping tacrolimus.

## Data Availability

The supporting for this report is available on request from the corresponding author.

## Abbreviations

WBC: White blood cell
Hb: Hemoglobin
Plt: Platelet
AST: Aspartate aminotransferase
ALT: Alanine transaminase
ALP: Alkaline phosphatase
HBV: Hepatitis B virus
HCV: Hepatitis C virus
HAV: Hepatitis A virus
CMV: Cytomegalovirus
COVID-19: Coronavirus disease- 2019
INR: International normalized ratio
PTT: Partial thromboplastin time
CNI: Calcineurin inhibitor
VOD: Venous occlusive disease
